# Complete genome sequence of *Stenotrophomonas* sp. strain C-A, isolated from contaminated *Pseudomonas putida* cultures

**DOI:** 10.1128/mra.00054-26

**Published:** 2026-05-18

**Authors:** Marissa N. Roghair Stroud, Larry J. Halverson

**Affiliations:** 1 Department of Plant Pathology, Entomology, and Microbiology, Iowa State University 1177, Ames, Iowa, USA; 2 Ames National Laboratory, U.S. Department of Energy, Iowa State University 1177, Ames, Iowa, USA; California State University San Marcos, San Marcos, California, USA

**Keywords:** genome, cell-cell interaction, *Stenotrophomonas*, *Pseudomonas putida*, biofilms

## Abstract

We report the complete *de novo* genome assembly of *Stenotrophomonas* sp. strain C-A, isolated from a contaminated culture of *Pseudomonas putida* KT2440. The genome’s annotation may inform how the two organisms interact with one another in mixed culture, and how growth state influences those interactions.

## ANNOUNCEMENT

We isolated *Stenotrophomonas* sp. strain C-A from a contaminated culture of *Pseudomonas putida* KT2440 (proposed to be named *P. alloputida* [1, 2]) used for planktonic and biofilm experiments in Reasoner’s 2A (R2A) broth. In these studies, we noted the appearance of a second colony morphology when dilution plating and counting colony-forming units. This second colony type was slower-growing, yellowish, and sticky, becoming detectable 24 h after KT2440 colonies were visible. To isolate *Stenotrophomonas* sp. C-A, the contaminated KT2440 culture was grown in R2A broth overnight, then diluted and plated onto Luria agar (LA), which showed two different colony morphologies. Multiple colonies of C-A were isolated and streaked for purity, all exhibiting identical growth properties. C-A grows well on LA, R2A, or ½-strength tryptic soy agar mediums, at both room temperature and 30°C.

*Stenotrophomonas* sp. C-A was grown overnight in LB, washed twice with 1 mM phosphate buffer, and resuspended in Zymo 1× DNA/RNA Shield. This was sent to Plasmidsaurus, who performed DNA isolation, library preparation, and sequencing. DNA was isolated using the Zymo Quick-DNA Miniprep Plus Kit. The library was prepared without amplification using the Nanopore Rapid Barcoding Kit 96 V14. This was sequenced with a primer-free protocol, using R10.4.1 flow cells on a PromethION P24 device, and Dorado v4.3 Super Accurate base-calling model. Read quality control was assessed with a minimum *Q*-score of 10, and adapter sequences were trimmed using MinKNOW.

Raw fastq files were used for *de novo* genome assembly using a pipeline developed in our lab (3), using default parameters except where otherwise noted. SeqKit v2.10.1 (4) was used to determine the number of raw reads (155,471) and *N*_50_ (11,539 bp). Reads were filtered using Chopper 0.11.0 (--quality 9 --minlength 1000) (5). Four different assembly programs were used to generate genome assemblies: Flye 2.9.6-b1802 (6), Raven 1.8.3 (7), Miniasm 0.3-r179 (8) with Minipolish v0.2.0 (9), and NextDenovo v.2.5.2 (10). A consensus assembly was generated using Trycycler v0.5.6 (11), polished with Medaka v2.0.1, and rotated to start at *dnaA* using Circlator v1.5.5 (12). The final genome was submitted to NCBI for annotation with the Prokaryotic Genome Annotation Pipeline (PGAP v6.10) (13). Completeness and contamination were assessed with EvalG, CheckM, and EvalCon (14, 15), accessed through the Bacterial and Viral Bioinformatics Resource Center Genome Annotation Service (16). The organism was identified as the genus *Stenotrophomonas* but did not match any known species based on a Type Strain Genome Server (17) analysis using default parameters; the closest related organism by genome phylogeny was *Stenotrophomonas geniculata* ATCC 19374 (BioSample: SAMN04207799).

The final assembled genome is 4,770,670 bp with 203.55× coverage and a GC content of 66.24%. There is only one contig (*N*_50_ = 4,770,670). There are 4,411 predicted genes and 4,318 predicted CDS in the PGAP annotation. The genome is estimated to have 100% completeness and 0% contamination.

## Data Availability

This whole-genome sequencing project has been deposited at NCBI under BioProject number PRJNA1373328 and BioSample number SAMN53651247. The assembled genome can be found with accession number CM132916.1. The raw reads have been deposited to NCBI’s SRA under accession number SRS27304381.
